# The potential health benefits of dietary natural plant products in age related eye diseases

**DOI:** 10.1016/j.heliyon.2020.e04408

**Published:** 2020-07-10

**Authors:** Eleazar Uchenna Ikonne, Victor Okezie Ikpeazu, Eziuche Amadike Ugbogu

**Affiliations:** a Department of Optometry, Abia State University, P.M.B 2000, Uturu, Abia State, Nigeria; b Department of Biochemistry, Abia State University, P.M.B 2000, Uturu, Abia State, Nigeria

**Keywords:** Natural plant products, Oxidative stress, Antioxidant, Anti-inflammatory, Eye diseases, Food science, Agricultural science, Biological sciences, Veterinary medicine, Health sciences

## Abstract

In the past decade, there has been a tremendous increase in the number of cases of age-related eye diseases such as age-related macular degeneration (AMD), cataract, diabetic retinopathy and glaucoma. These diseases are the leading causes of visual impairment and blindness all over the world and are associated with many pathological risk factors such as aging, pollution, high levels of glucose (hyperglycaemia), high metabolic rates, and light exposure. These risk factors lead to the generation of uncontrollable reactive oxygen species (ROS), which causes oxidative stress. Oxidative stress plays a crucial role in the pathogenesis of age-related eye diseases through the activation of nuclear factor kappa B (NF-κB), vascular endothelial growth factor (VEGF), and lipid peroxidation, which leads to the production of inflammatory cytokines, angiogenesis, protein and DNA damages, apoptosis that causes macular degeneration (AMD), cataract, diabetic retinopathy and glaucoma. This review provides updated information on the beneficial effects of dietary natural plant products (DPNPs) against age-related eye diseases. In this review, supplementation of DPNPs demonstrated preventive and therapeutic effects on people at risk of or with age-related eye diseases due to their capacity to scavenge free radicals, ameliorate inflammatory molecules, neutralize the oxidation reaction that occurs in photoreceptor cells, decrease vascular endothelial growth factor and the blood-retinal barrier and increase the antioxidant defence system. However, further experiments and clinical trials are required to establish the daily doses of DPNPs that will safely and effectively prevent age-related eye diseases.

## 1 Introduction

The human eye is a sensitive, delicate and sensory organ that is used for light perception and vision. In the eye, oxidative stress caused by photooxidation and high metabolic rate leads to progressive and irreversible loss of vision in age-related eye diseases such as age-related macular degeneration (AMD), diabetic retinopathy, glaucoma and cataract (Rauf et al., 2017; Bungau et al., 2019). Although eye diseases are associated with many pathological factors such as aging, angiogenesis, diabetes, genetic predisposition, ischemia, inflammation, oxidative stress and tumorigenesis, oxidative stress plays a crucial role in pathogenesis of age-related eye diseases (Williams, 2008; Tsai et al., 2010). Oxidative stress is a common denominator of toxicity that results from an imbalance between oxidant and antioxidant systems (Abdel-Daim et al., 2019). It is a major player in the pathogenesis of inflammatory disorders, cancer, diabetes, neurodegenerative and cardiovascular diseases.

Loss of vision caused by age-related eye diseases has compromising effects on quality of life and may also result in an economic burden on the individual, the family and society (Kim et al. 2010). In 2010, WHO reported that 253 million individuals are living with visual impairment, 39 million of whom are blind, and it is estimated that by 2050 that the number will triple (WHO, 2012; Bourne et al., 2017). Köberlein et al. (2013) predicted that the economic burden of visual impairment will be more than $70 billion in developed countries.

Unfortunately, the pathogenic process of these age-related eye diseases is complex and depends on many unclear factors. Again, most of these age-related eye diseases are diagnosed during their late or advanced stages, and in most cases the effective treatments are not readily available (Rhone and Basu, 2008; Bungau et al., 2019). Today, the preventive, diagnostic and treatment measures of age-related eye diseases have become one of the top global health concerns. The health complications and economic implications of these age-related eye diseases in humans have propelled many researchers to develop a keen interest on various strategies, including the use of plant-based natural products as a therapy to minimize these diseases and their associated problems.

Plant-based natural products are plant metabolites found in fruits and vegetables that play vital roles in plant protection against herbivores. They do not only possess phytopotentials for growth and reproduction in humans, but are also used in the management and treatment of several diseases (Bodas et al. 2008; Ugbogu et al., 2019).

Many researchers have experimentally shown substantial scientific evidence that consumption of these plant-based natural products has the potential to prevent vision loss and reverse visual impairment (Cho et al., 2004; Tang et al., 2011), due to their anti-inflammatory and antioxidant activities (de la Cámara et al., 2013). Antioxidants are naturally abundant in dietary sources such as fruits and vegetables (Yeung et al. 2019). Many researchers have reported that consumption of diet-based antioxidants ameliorate cellular oxidative stress/damage, and therefore have the potential to prevent cancer, diabetes, neurodegenerative and cardiovascular diseases (Pandey and Rizvi, 2009; Abdel-Daim et al. 2018, 2019). This review will evaluate the roles of selected plant-based natural products such as polyphenols, carotenoids, provitamin A, vitamins C and E in the prevention and treatment of age-related eye diseases like age-related macular degeneration (AMD), diabetic retinopathy, glaucoma and cataract.

## 2 Materials used for this study

In this study, relevant materials were obtained using only online database such as Pubmed, sciencedirect and springer. The search terms include; effects of dietary plant products on age-related eye diseases, effects of polyphenols or carotenoids or vitamin A, C, E on cataract or glaucoma or diabetic retinopathy or age-related macular degeneration. The effects of dietary plant natural products such as curcumin, resveratrol, quercetin, caffeine, epigallocatechin gallate, and lycopene on age-related eye diseases were also searched specifically for each dietary plant natural products. Only published papers written in English language were selected and used in this study.

## 3 Age-related eye diseases

The studied age-related eye diseases are age-related macular degeneration (AMD), diabetic retinopathy, glaucoma and cataract.

### 3.1 Age-related macular degeneration (AMD)

AMD is a chronic retinal disease caused by the progressive loss of photoreceptors or macular pigment (Vujosevic et al., 2017). It is one of the leading causes of vision loss or vision impairment among elderly people above 50 years of age (Mathenge et al., 2012). The major risk factors are age, arteriosclerosis, family history, hypertension, hypercholesterolemia, obesity and smoking (Kim et al. 2010). There are basically two types of AMD; wet (neovascular) and dry (atrophic) AMD (Kaarniranta et al., 2015). In wet (neovascular) AMD, angiogenic cytokines like vascular endothelial growth factor A (VEGF-A) causes choroidal neovascularization (Salminen et al., 2010). It is characterized by the abnormal growth of new blood vessels beneath the retina. This compromises the central vision acuity by damaging the macula and reduces macular density (Chiu et al., 2019). It affects 10–15% of individuals and results into 90% blindness in AMD patients (Abdel-Aal et al., 2013). In dry (atrophic) AMD, there are white or yellowish deposits on the retina and also loss of retinal pigment. It affects 80–90% of the people (Van Lookeren Campagne et al., 2014; Chiu et al., 2019). AMD can also be classified into early-stage, intermediate-stage and late-stage (Chiu et al., 2019). In early-stage AMD, no symptom or vision loss is observed in the patients. In intermediate-stage AMD, slight vision loss might be observed in the patients without symptoms while in the late stage of AMD the patients experience visual impairment (Vujosevic et al., 2017). The major treatment for wet AMD is the use of intravitreous anti-VEGF with monoclonal antibodies or aptamers (Salminen et al., 2010).

### 3.2 Glaucoma

Glaucoma is a heterogeneous disease characterized by a progressive and irreversible loss of retinal ganglion cells (RGC) neurodegeneration (Geyera and Levo, 2020). It is an optic neuropathy that causes vision loss and irreversible blindness and can be referred to as “the sneak thief of sight” (Loskutova et al., 2019). Worldwide, glaucoma is ranked second most common cause of blindness after the cataract and the highest cause of preventable visual disability (Quigley and Broman, 2006; Butt et al., 2016). Globally, over 70 million individuals suffer from glaucoma with about 10% bilaterally blind (Quigley and Broman, 2006). The main risk factors of glaucoma include elevated intraocular pressure (IOP) above 22 mmHg, aging, high myopia, vascular dysregulation and family history (Loskutova et al., 2019). However, elevated intraocular pressure (IOP) is the major risk factor for glaucoma. Glaucoma affects mainly the middle-aged and elderly individuals. The two major types of glaucoma are the open angle and the angle closure glaucoma. Open angle glaucoma develops very slowly while close angle glaucoma occurs unexpectedly and its signs and symptoms include slight vision loss and pains (Yadav et al., 2019). The other kinds of glaucoma are normal tension glaucoma, which is rare and causes optic nerve damage, pigmentary glaucoma that occurs in early or middle adulthood and causes a rise in eye pressure because the iris pigment cells disperse in the eye (Kountouras et al., 2004), and the uveitic glaucoma that occurs because of changes in aqueous production and its outflow, thus causing a change in the structure of the anterior chamber angle (Yadav et al., 2019). Glaucoma can be managed or treated with laser treatment, oral drugs, reducing the intraocular pressure (IOP) using eye drops or through surgery (Mandelcorn and Gupta, 2009; Prum et al., 2016).

### 3.3 Cataract

Cataract is an age-related eye disease that is caused by the opacity of the lens or loss of transparency due to long term exposure of light that decomposes components of the lens (Chu and Pang, 2014). It is mainly caused by structural changes in lens protein known as crystallin (Chu and Pang, 2014). Worldwide, it is the most prevalent age-related eye disease that causes reversible blindness (Zhu et al., 2017; Thrimawithana et al., 2018).

In middle-income and low-income countries cataract is the leading cause of blindness (Liu et al., 2017) and 95 million individuals worldwide suffer cataract (Liu et al., 2017). WHO reported that cataract leads to 51% of blindness and 33% of visual impairment globally (Mario, 2012). Bourne et al. (2017) estimated that un-operated cataract leads to 35% blindness and 25% vision impairment. The main risk factors of cataracts are aging, diabetes mellitus, eye injury, exposure to ultraviolet light, hypertension, smoking, steroid use, trauma and family history (Beebe et al., 2010; Zigler and Datiles, 2011; Roberts, 2011). The symptoms of cataracts include; colour disturbance, decreased contrast sensitivity, glare and impaired vision (Song et al., 2014).

Clinically, cataracts can be classified into three types; cortical cataracts, nuclear cataracts and posterior subcapsular cataracts. However, it is important to note that the three types can associate with each other when left untreated as they progress to cause complete lens opacification (Liu et al., 2017). The cortical cataract is often wedged-shaped that originates from lens cortex and extend to the centre of the lens. It has links with diabetes mellitus. Nuclear cataracts associate with increase in age and degenerates by its progression and colour. Subcapsular cataract is a plaque-like opacity that emanates from the axial posterior cortical layer (Sparrow et al., 1986; Liu et al., 2017). The most effective way to treat cataracts is through surgery, although it is expensive and in most countries people cannot afford the treatment.

### 3.4 Diabetic retinopathy

The diabetic retinopathy is a metabolic disorder that alters the photoreceptors and the blood vessels of the retina. It is one of the major complications of type 1 and 2 diabetes (López-Malo et al., 2020). Worldwide, diabetic retinopathy is a leading cause of blindness, especially among people that are yet to reach their retirement age (Lopez-Galvez et al., 2014), and has been estimated to affect over 140 million people (Yau et al., 2012). Oxidative stress is the major risk factor for the development of diabetic retinopathy and its critical phases such as diabetic macular edema and proliferative diabetic retinopathy (Lopez-Galvez et al., 2014). Oxidative stress occurs because of imbalance between anti-oxidative mechanisms and reactive oxygen species, and this causes an increase in glucose levels (Tesfaye et al., 2005). Diabetic retinopathy is characterised by glaring retinal-blood barrier and vascular disturbances (Bungau et al., 2019). In diabetic retinopathy, hyperglycemia triggers complex metabolic abnormalities in the retina that produces reactive oxygen species that induces oxidative stress in the retina (Li et al., 2017). The induced oxidative stress leads to retinal lesions and causes angiogenesis, endothelial cell dysfunction and periocitary (Li et al., 2017). Diabetic retinopathy is mainly grouped into two; proliferative retinopathy and nonproliferative retinopathy (Parsamanesh et al., 2018). In proliferative retinopathy, laser surgery is used to control leakage of fluids by shrinking the abnormal blood vessels, however in severe bleeding a vitrectomy is employed to evacuate the blood from the centre of the eye (Fong et al., 2003; Yau et al., 2012). Nonproliferative retinopathy can be subdivided into three stages; mild, moderate, and severe. It is characterised by alteration of intra-retinal microvasculature (Frank, 2015). No treatment is needed for the nonproliferative retinopathy; however, patients need to control their blood pressure, blood sugar and blood cholesterol levels (Fong et al., 2003). When there are apparent pathological signs such as angiogenesis of the retina, fluid accumulation, hemorrhage, vascular permeability and vitreous fibrotic response, nonproliferative retinopathy may progress to proliferative retinopathy (Scholl et al., 2010).

## 4 Protective effects of dietary natural plant products against age-related eye diseases

**Figure 2:**
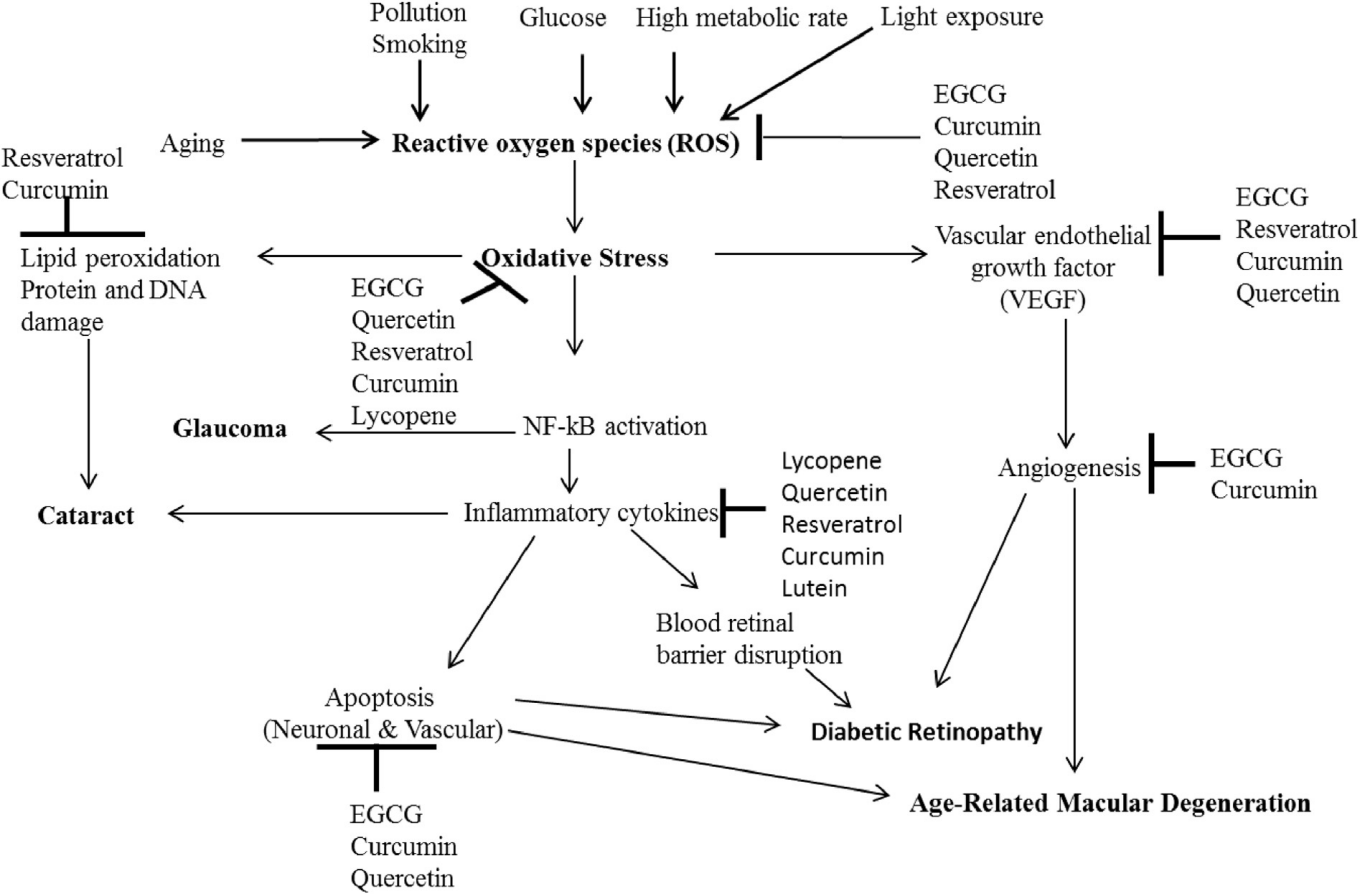
Protective effects of dietary natural plant products against age-related eye diseases.

Age-related eye diseases are caused by many risk factors such as aging, air pollution, smoking, high levels of glucose (hyperglycemia), high metabolic rates and light exposure (Chiu et al., 2019). These risk factors lead to generation of uncontrollable reactive oxygen species (ROS) that causes oxidative stress. Oxidative stress plays a crucial role in the pathogenesis of age-related eye diseases by activating nuclear factor kappa B (NF-kB), vascular endothelial growth factor (VEGF), and lipid peroxidation which leads to the production of inflammatory cytokines, angiogenesis, protein and DNA damage, and apoptosis (Figure 2). These result to chronic age-related eye diseases such as; age-related macular degeneration (AMD)- caused by the damage in the retinal pigment epithelium (RPE) and photoreceptors; cataracts-caused by damage to the protein, DNA and membrane fibre which lead to loss of lens transparency due to imbalance in electrolytes; diabetic retinopathy-caused by endothelial cell dysfunction, disruption of blood retinal barrier; glaucoma-caused by the alteration of human trabecular mesh-work, this leads to the drainage of the aqueous humour and an increase in intraocular pressure (IOP) (Bungau et al., 2019). Scientific evidences have shown that dietary plant natural products have potential beneficial effects in the prevention, management and treatment of age-related eye diseases (Abdel-Daim et al., 2019). The potential mechanisms by which the dietary plant natural products protect against age-related eye diseases are shown in Figure 2. Supplementation of dietary plant natural products has demonstrated preventive and therapeutic effects because of its capacity to scavenge free radicals and reduce enzymes involved in reactive oxygen species production. It neutralizes the oxidation reaction that occurs in photoreceptor cells and upregulate antioxidant defence system. They have also shown their capacity to reduce opacification of the suppressed lens and apoptosis of the retinal pigment epithelium, inhibition of the inflammatory markers and blood-retinal barrier and improve ocular blood flow (Xu et al., 2017). The beneficial effects of the studied dietary plant natural products (Table 1 and Figure 2) are summarized below;

**Table 1:** Effects of plant derived natural products on eye-related age diseases.

Plant derived natural products	Doses	Experimental model	Observations	Effects on eye diseases	References
Epigallocatechin gallate	50 μM	Human lens epithelial cells	Resists H_2_O_2_-induced apoptosis, and ROS, and protects against mitochondrial dysfunction.	Inhibits the progression of cataracts.	Yao et al. (2008)
Epigallocatechin gallate	1–50 mM	Human RPE cell line ARPE-19	Inhibits ocular angiogenesis and vascular permeability.	Prevents age-related macular degeneration (AMD) and diabetic retinopathy.	Lee et al. (2014)
Epigallocatechin gallate	20 and 40 mM	Human retinal endothelial cell	Inhibits expression of vascular endothelial growth factor (VEGF) and reduces negative impact of high glucose concentration on the cell viability.	Prevents diabetic retinopathy	Zhang et al. (2016)
Epigallocatechin gallate	1–200 μM	Sprague-Dawley rats	Inhibits cell proliferation and reduces vascular leakage and permeability in VEGF.	Prevents ocular angiogenic diseases, e.g. age-related macular degeneration (AMD) and diabetic retinopathy.	Lee et al. (2014)
Epigallocatechin gallate		Human	Positively influence inner retinal function.	Inhibits glaucomatous damage.	Falsini et al. (2009)
Epigallocatechin gallate	Intraperitoneal (25 mg/kg) Intraocular (5 μL of 200 μM)	Wistar rats	Protects retinal neurons from oxidative stress and ischemia/reperfusion, reduces the apoptosis to retinal ganglion cells.	Prevents glaucoma.	Zhang et al. (2007)
Quercetin	50 μM	Cultured human RPE cells	Protects RPE cells from oxidative damage and cellular senescence.	Prevents age-related macular degeneration (AMD).	Kook et al. (2008)
Quercetin	50 μM	Cultured human RPE cells (ARPE-19)	Protects human RPE cells from oxidative stress via the inhibition of proinflammatory molecules.	Prevents age-related macular degeneration (AMD).	Cao et al. (2010)
Quercetin	10 μM	Rat lens (Wistar rats)	Increases neurotrophic factors and inhibits cytochrome c and caspase-3 levels.	Prevents cataract.	Sanderson et al. (1999)
Quercetin	50 mg/body weight/kg	Sprague-Dawley rats	Decreases photooxidative damage in the retina and mediates cytoprotection against light-induced photoreceptor cell degeneration in rats.	Inhibits age-related eye diseases.	Koyama et al. (2019)
Quercetin	50 mg/kg/day	Diabetic rat retina	Protects the neuronal damage, ameliorates neurotrophic factors and inhibits the apoptosis of neurons	Prevents neurodegeneration in diabetic retinopathy.	Ola et al. (2017)
Quercetin and chlorogenic acid	33.63 mg/kg/day	Pigmented rabbits	Alleviates retinal degeneration.	Prevents AMD.	Wang et al. (2017)
Resveratrol	40 mg/kg	Sprague-Dawley rat lens	Suppresses selenite-induced oxidative stress and cataract formation in rats.	Inhibits selenite-induced cataractogenesis.	Doganay et al. (2006)
Resveratrol	5 mg/kg/day	Streptozotocin-induced diabetic Wistar rats	Suppresses oxidative stress.	Prevents diabetic retinopathy.	Soufi et al. (2012)
Resveratrol	20 mg/kg	Streptozotocin-induced diabetic C57BL/6 mice	Decreases vascular lesions and VEGF induction.	Prevents diabetic retinopathy.	Kim et al. (2012)
Resveratrol	10 mg/kg	Streptozotocin-induced diabetic Wistar rats	Suppresses the expression of eNOS actively involved in inflammation.	Prevents diabetic retinopathy.	Yar et al. (2012)
Resveratrol	5 mg/kg	Streptozotocin-induced diabetic Wistar rats	Inhibits inflammation.	Prevents diabetic retinopathy.	Ghadiri et al. (2015)
Resveratrol	5 and 10 mg/kg/day	Diabetic rat retina	Alleviates hyperglycemia and weight loss.	Prevents diabetic retinopathy.	Zeng et al. (2016)
Resveratrol	10, 20, and 40 μmol/L	Human lens epithelial cells	Inhibits oxidative stress.	Prevents cataract.	Zeng et al. (2016)
Resveratrol	5 and 10 mg/kg/day	High-glucose culture Müller-treated cells	Prevents production of intracellular reactive oxygen species (iROS) and inflammatory markers.	Prevents diabetic retinopathy.	Luna et al. (2009)
Zeaxanthin	0.02% or 0.1%	Age-matched normal rats	Inhibits the development of retinopathy in diabetics.	Prevents diabetic retinopathy.	Kowluru et al. (2008)
Lutein	0.5 mg/kg	Streptozotocin-induced diabetic rats	Prevents the diabetes-induced decrease in glutathione content.	Prevents cataract.	Arnal et al. (2009)
Curcumin	50 μM	Rat organ cultured lens	Suppresses oxidative stress, prevents uncontrolled generation of free radicals, and inhibits iNOS expression.	Suppresses cataract formation.	Manikandan et al. (2009)
Curcumin	75 mg/kg	Wistar rats	Prevents selenium-induced Ca^2+^ -ATPase activation.	Inhibits cataract.	Manikandan et al., 2010a, Manikandan et al., 2010b
Curcumin	0.005% (w/w)	Wistar rats	Alleviates naphthalene-induced cataract.	Prevents cataract.	Pandya et al. (2000)
Curcumin	0.5 g/kg	Rats	Reduces DNA damage by decreasing the NF- κB activation, and increases antioxidant capacity.	Prevents diabetic retinopathy.	Kowluru, and Kanwar (2007)
Curcumin	1 g/kg	Wistar albino rats	Elevates antioxidant defence system, decreases retina expression of proinflammatory cytokines.	Inhibits diabetic retinopathy.	Gupta et al. (2011)
Curcumin	80 mg/kg	Sprague-Dawley rat	Decreases retinal glutamine and oxidative stress.	Prevents diabetic retinopathy.	Zuo et al. (2013)
Curcumin	100 and 200 mg/kg/day	Wistar albino rats	Restores retinal antioxidant capacity, decreases retina expression of proinflammatory cytokines	Prevents diabetic retinopathy.	Yang et al. (2018)
Curcumin	75 mg/kg	Wistar rats	Increases the levels of superoxide dismutase, catalase and GSH.	Prevents cataract formation.	Manikandan et al. (2010b)
β-carotene, β-cryptoxathin, lutein, zeaxanthin, and lycopene	-	Human	Participants with the highest self-reported dietary intake of lutein and zeaxanthin were inversely associated with advanced age-related macular degeneration (AMD).	Inhibits AMD.	Delcourt et al. (2006)
Vitamin A, vitamin C, and vitamin E	-	Human	Dietary intake of a mixture of vitamin A, vitamin C, and vitamin E had a larger effect on the reduction of AMD risk than the individual vitamin.	Inhibits AMD.	SanGiovanni et al. (2007)
Vitamin A, vitamin C, and vitamin E	-	Human	Low dietary intake of vitamin C and vitamin E was associated with reduced risk of neovascular AMD.	Inhibits AMD.	Aoki et al. (2016)
Vitamin C and vitamin E	-	Human	No effect on vitamin status and neovascular AMD.	No effect on AMD.	Eye Disease Case-Control Study Group (1993)
Provitamin A, β-carotene, vitamin C, and vitamin E	-	Human	High intake of β-carotene, vitamin C, and vitamin E reduce the risk of neovascular AMD.	Inhibits AMD.	Zampatti et al., (2014)
Caffeine	50–250 mg/day	Human	Increases antioxidant and bioenergetic effect on the lens.	Inhibits Cataract.	Varma (2016)
Caffeine	72 mM	Sprague Dawley rats	Inhibits formation of galactose cataract.	Protects diabetic cataract.	Varma et al. (2010)
Caffeine	20 mg/kg	Wistar rats	Decreases the activities of SOD, CAT and MDA.	Inhibits cataract.	Kaczmarczyk-Sedlak et al. (2019)
Caffeine	0.2 mL/day	Sprague Dawley rats	Reduces cataract formation.	Prevents cataract.	Ishimori et al. (2017)
Lycopene	4 mg/kg	Wistar rats	Prevents inflammation and oxidative stress on the eye tissues.	Inhibits diabetic retinopathy.	Icel et al. (2019)
Lycopene	200 μg/kg	Wistar rats	Delays the onset and the progress of galactose-induced cataract in *in vivo* study.	Inhibits cataract.	Gupta et al. (2003)

Caffeine-decreases the activities of SOD, CAT and MDA, and increases antioxidant activities.

Curcumin-inhibits lipid peroxidation, reactive oxygen species and vascular endothelial growth factor, suppresses oxidative stress, decreases pro-inflammatory cytokines, and reduces DNA damage by decreasing NF-kB activation, and increase antioxidant enzymes.

Epigallocatechin gallate (EGCG)- inhibits reactive oxygen species, angiogenesis, VEGF, protects against mitochondrial dysfunction, reduces vascular leakage and permeability in VEGF, and also reduces apoptosis of retinal ganglion cells.

Lycopene-prevents inflammation and oxidative stress.

Quercetin-inhibits ROS, VEGF, pro-inflammatory molecules and apoptosis of the neurons, and protects RPE cells.

Reseveratrol-inhibits oxidative stress, reactive oxygen species, vascular endothelial growth factor, lipid peroxidation, reduces inflammatory molecules and increases glutathione (GSH).

## 5 Effects of dietary natural plant products against age-related eye diseases

### 5.1 Curcumin

Curcumin (1,7-bis-(4-hydroxy-3-methoxyphenyl)-1,6-heptadiene-3,5dione) is a plant-derived lipophilic polyphenol obtained from the turmeric (*Curcuma longa* roots) (Bansal et al., 2018) Figure 1. It is one of the DPNPs with wide pharmacological properties such as antimicrobial, anti-inflammatory and antioxidative, antimutagenic and anticancer activities (Davis et al., 2018; Bansal et al., 2018; Radomska-Leśniewska et al., 2019). It has the potentials to effectively inhibit lipid peroxidation, reactive oxygen species, decrease inflammatory cytokines, suppress oxidative stress, and increase antioxidant enzymes in age-related eye diseases (Figure 2) (Suryanarayana et al., 2005; Xu et al., 2017). Many researchers have documented positive effects of curcumin against age-related eye diseases such as cataract (Pandya et al., 2000; Manikandan et al., 2010a) and diabetic retinopathy (Zuo et al., 2013; Yang et al., 2018). The work of Mandal et al. (2009) reported that supplementation of 0.2% curcumin in rat-diets for two weeks inhibited NF-kΒ activation and downregulated inflammatory genes which leads to retinal neuroprotection. In a human retinal cell experiment, incubation of the cells with 15 μM curcumin caused cytoprotective effects by inhibiting reactive oxygen species (Woo et al., 2012). Mrudula et al. (2007) reported that the addition of 0.01% or 0.5% of curcumin in mice diets for 8 weeks had an inhibitory effect on VEGF expression while 1 g/kg of curcumin administered to mice for 16 weeks decreased GSH, SOD, catalase, TNF-α, and VEGF in diabetic retinopathy induced mice. Kowluru and Kanwar (2007) observed that addition of 0.5 g/kg of curcumin in rats inhibited NF-kB activation and increased antioxidant activity thus leading to prevention of diabetic retinopathy. Gupta et al. (2011) reported that inclusion of curcumin at the concentration of 1 g/kg in Wistar rats suppressed the expression of pro-inflammatory cytokines and elevated antioxidant defence system that resulted in inhibition of diabetic retinopathy. Zuo et al. (2013) demonstrated that supplementation of 80 mg/kg curcumin in Sprague-Dawley rats scavenged the oxidative stress, reduced retinal glutamine and prevented diabetic retinopathy. Restoration of antioxidant activities and decreased retina pro-inflammatory cytokines were observed in rats treated with 100–200 mg/kg of curcumin (Yang et al., 2018). Suryanarayana et al. (2003) reported that 0.002% curcumin delayed cataract formation. Supplementation of 0.005% of curcumin in rats alleviated naphthalene-induced cataract (Pandya et al., 2000). Manikandan et al. (2010a, b) observed that the administration of 75 mg/kg of curcumin in Wistar rats resulted in the inhibition of selenium-induced Ca^2+^-ATPase activation Manikandan et al. (2010a), increased superoxide dismutase, catalase, and GSH and prevented cataract formation (Table 1).

**Figure 1:**
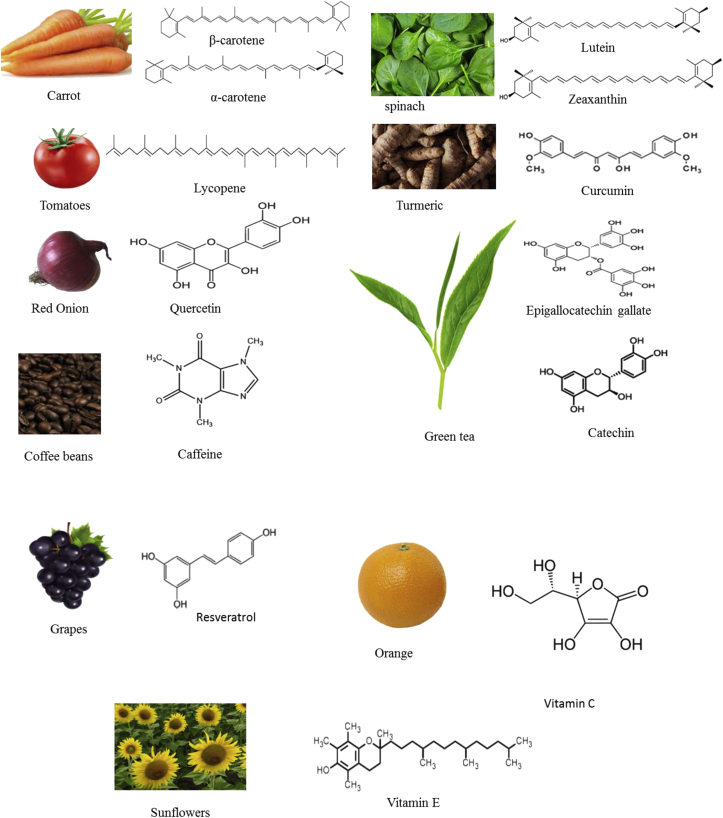
Representative of natural plant products and their dietary sources.

### 5.2 Quercetin

Quercetin (2-(3,4-dihydroxyphenyl)-3,5,7-trihydroxychromen-4-one) is one of the plant polyphenols from flavonoids that is mostly found in red onion, apples, tomato, blueberries, red grapes, broccoli and citrus fruits (Rice-Evans et al., 1995; Bansal et al., 2018) Figure 1. It has antioxidant and anti-inflammatory effects, and is widely used in the treatment of several diseases like hay fever, asthma, viral infections, peptic ulcer, diabetes, gout, eye diseases and schizophrenia (Bansal et al., 2018). It has the capacity to inhibit ROS, VEGF, pro-inflammatory molecules, and apoptosis of the neurons, and protect RPE cells in age-related eye diseases (Figure 2). Documented scientific evidence has shown that quercetin inhibits AMD (Chen et al., 2008), glaucoma (Ishii et al., 2003) and cataract (Stefek and Karasu, 2011) in animal and human studies. Ola et al. (2017) opined that 50 mg/kg of quercetin administered to diabetic rat-retina protected the rats against neuronal damage and inhibited apoptosis of the neurons, which prevented neurodegeneration in diabetic retinopathy. Kook et al. (2008) and Cao et al. (2010) in their different experiments observed that 50 μM of quercetin protected human RPE cells from oxidative stress, cellular senescence and also inhibited pro-inflammatory molecules, which resulted in the prevention of age-related macular degeneration (AMD). Koyama et al. (2019) reported that 50 mg/bodyweight/kg of quercetin in rat model experiment decreased photooxidative damage in the rat retina. However, in cataract model experiment, Sanderson et al. (1999) documented that 10 μM of quercetin increased neurotropic factors and inhibited cytochrome C and caspase-3 levels (Table 1).

### 5.3 Epigallocatechin gallate

Epigallocatechin gallate (EGCG) ([(2R,3R)-5,7-dihydroxy-2-(3,4,5-trihydroxyphenyl)-3,4-dihydro-2H-chromen-3-yl] 3,4,5-trihydroxybenzoate) is the most abundant polyphenol in catechin. It is mainly found in green tea (Gan et al., 2018; Bansal et al., 2018) Figure 1. It has antiangiogenic, antiatherogenic, anticancer, anti-inflammatory and antioxidative activities (Chen et al., 2011). In age-related eye diseases, epigallocatechin gallate inhibits reactive oxygen species, angiogenesis, VEGF, apoptosis of retinal ganglion cells and protects against mitochondrial dysfunction (Figure 2). Yao et al. (2008) reported that administration of 50 μM EGCG in human lens epithelial cells reduced ROS and mitochondrial dysfunction thus leading to a reduction in cataract formation. Lee et al. (2014) observed that the inclusion of 1–50 mM EGCG in human RPE cell line ARPE-19 inhibited angiogenesis and vascular permeability, which displayed preventive effect against AMD and diabetic retinopathy. Similar observations were reported by Alex et al. (2010) and Chan et al. (2010). Zhang et al. (2008) and Costa et al. (2008) established that oral administration of EGCG prevented photoreceptor cell death in light-induced retinal neuronal death. Zhang and Osborne (2006) observed protective effect of EGCG on the retinal photoreceptors. Supplementation of EGCG inhibited the expression of VEGF, reduced negative impact of hyperglycemia on the viability of cells and resulted in inhibition of diabetic retinopathy (Zhang et al., 2016). In addition, Zhang et al. (2007) reported that EGCG had protective effects on retinal neurons against oxidative stress or ischemia/reperfusion insult, which leads to the prevention of glaucoma (Table 1).

### 5.4 Resveratrol

Resveratrol RSV (3,5,4ʹ-trihydroxystilbene) is a low molecular weight polyphenol found in berries, grapes, peanuts, jackfruit and cranberry (Keylor et al., 2015; Gambini et al., 2015) Figure 1. It possesses antioxidant, antibacterial, anticancer and antifungal properties (Salehi et al., 2018). In age-related eye diseases, supplementation of resveratrol inhibits oxidative stress, reactive oxygen species, vascular endothelial growth factor, lipid peroxidation, reduces inflammatory molecules and increases GSH in animal and human experimental models (Kim et al., 2012; Yar et al., 2012).

Supplementation of 5 mg/kg/day of resveratrol in streptozotocin-induced diabetic rats significantly suppressed oxidative stress, eNOS activity in the blood and the retina, and decreased hyperglycemia (Soufi et al., 2012). Kim et al. (2012) reported that administration of 20 mg/kg in diabetic rats inhibited VEGF, vessel leakage, and pericyte loss. Similarly, many researchers demonstrated that resveratrol treatment prevented the destruction of neuronal cell and vascular hyper permeability in diabetes-induced cells (Yar et al., 2012; Ghadiri Soufi et al., 2015; Zeng et al., 2016) and protected RPE cells (Anekonda, and Adamus, 2008). An inhibitory effect of resveratrol on selenite-induced cataract was observed by Doganay et al. (2006). In their study, they reported that 40 mg/kg of resveratrol administered to Sprague-Dawley rat lens suppressed oxidative stress, reduced lipid peroxidation and prevented cataract formation in rats. It has been observed that supplementation of resveratrol had the potentials to significantly reduce glaucoma markers, and inhibits proinflammatory markers and apoptotic effects in trabecular mesh-work cells (Luna et al., 2009; Bola et al., 2014). The study of Lu et al. (2006) demonstrated that addition of resveratrol increased blood flow, which could result in the inhibition of the apoptotic optic nerve cells and prevents vessel damage in glaucoma patients (Table 1).

### 5.5 Lycopene

Lycopene is a naturally water-insoluble dietary carotenoid that is found in tomatoes, watermelon, pink grapefruit and papaya (Bansal et al., 2018) Figure 1. It has anticancer, antioxidant, anti-inflammatory neuroprotective and osteoprotective effects (Wei and Giovannucci, 2012; Yang et al., 2016). In age-related eye diseases, lycopene prevent inflammation, oxidative stress and angiogenesis (Yang et al., 2016). Yang et al. (2016) in their study observed protective effects of lycopene on retinal pigment epithelium in ARPE-19 cell against oxidative stress and inflammation. Lycopene scavenged oxidative stress in retinal tissues in diabetes-induced retinopathy (Li et al., 2010). In an *in vivo* study, oral administration of 200 μg/kg of lycopene in rats significantly delayed the onset and progression of galactose-induced cataract, and improved the antioxidant enzymes (GSH and malondialdehyde) (Gupta et al., 2003). Icel et al. (2019) reported that lycopene inhibited inflammation and oxidative stress, which results in amelioration of diabetic retinopathy in diabetes-induced optic neuropathy (Table 1).

### 5.6 Caffeine

Caffeine (1,3,7-trimethylxanthine) is a naturally occurring purine alkaloid in coffee that is consumed globally (Yoon and Danesh-Meyer, 2019). It is found mainly in *Coffea arabica* and *C. canephora* plants (Kronschlager et al., 2013) Figure 1. It is used in the treatment of bronchopulmonary dysplasia in premature infants, post-lumbar puncture headache, neonatal apnea and acute migraine (Yoon and Danesh-Meyer, 2019). In age-related eye diseases, caffeine has the potentials to decrease the activities of SOD, CAT and MDA (Kaczmarczyk-Sedlak et al., 2019), and increase antioxidant activities (Varma, 2016). Zhang et al. (2017) reported that caffeine had a protective effect against oxygen-induced retinopathy in mice. Varma et al. (2010) showed that supplementation of 72 mM of caffeine in rats resulted in the inhibition of cataract formation. Kaczmarczyk-Sedlak et al. (2019) demonstrated that the administration of 20 mg/kg caffeine decreased the activities of SOD, CAT and MDA, and ameliorate cataract formation. In human, Varma (2016) reported an increase in antioxidant activities, bioenergetic effects and reduced cataract formation Table 1. Ajayi and Ukwade, 2001 and Vera et al., (2019) posited that caffeine increases intraocular pressure (IOP). Chandrasekaran et al. (2005) reported that coffee intake elevates IOP in glaucoma patients. Chandra et al. (2011) reported that administration of caffeine into the eyes of patients does not affect the IOP and therefore suggest that caffeine has no significant effect on IOP in patients with glaucoma. Therefore, further research is required to establish the short and long-term effects of caffeine consumption on the IOP of glaucoma patients.

### 5.7 Lutein and zeaxanthin

Lutein and zeaxanthin are dietary carotenoid xanthophylls mostly found in spinach, carrots and red grapes (Chu and Pang, 2014) Figure 1. They are referred to as macular pigments because of their higher concentrations in human macula and retina (Bernstein et al., 2016). They have cardioprotective, neuroprotective, anti-inflammatory, anticancer and antioxidant properties (Abdel-Aal et al., 2013; VijayaPadma et al., 2014).

In human intervention study, supplementation of lutein and zeaxanthin significantly improved visual performances and prevented the progression of early AMD (Ma and Lin, 2010; Hammond et al., 2014). Epidemiological studies conducted by Delcourt et al. (2006) and Mares (2004) demonstrated that the administration of lutein and zeaxanthin had an increasing effect on plasma lutein and zeaxanthin levels, which resulted in significant inhibition of cataract and AMD. The meta-analysis study of Ma et al. (2012) revealed that higher intake of lutein and zeaxanthin reduced the risk of late AMD. In the animal studies, administration of zeaxanthin decreased retinal oxidative stress and pro-inflammatory cytokines (Kowluru et al., 2014), inhibited lipid peroxidation and prevented VEGF (Kowluru et al., 2008) in diabetic rats. Similarly, lutein had a protective effect on retina against diabetes-induced oxidative stress (Muriach et al., 2006) Table 1.

### 5.8 Vitamins C, E and provitamin A (α-carotene and β-carotene)

Vitamin C (ascorbic acid) is a water-soluble vitamin. It has high and effective antioxidant capacity that protects carbohydrates, proteins, lipids and nucleic acids from damage caused by free radical and reactive oxygen species (Zampatti et al., 2014). It is found mostly in fruits and vegetables such as orange, pineapple, lemon, lime, onion, cabbage and tomatoes (Andreatta and El-Sherbiny, 2014). It is beneficial and can help prevent age-related eye diseases due to its strong antioxidant activities. Weikel et al. (2014) reported that intake of 135 mg/day or blood concentrations of 6 μM of vitamin C decreased the risk of nuclear cataract by 40%. Another study revealed that vitamin C can only prevent nuclear cataract in humans (Tan et al., 2008). Vitamin E is a fat-soluble vitamin also known as tocopherol that consists of four tocotrienols (alpha, beta, gamma, and delta tocopherols). The most studied Vitamin E is the alpha tocopherol (Brigelius-Flohe and Traber, 1999). Vitamin E is found mostly in sunflower, soybean, peanut, and pumpkin. It has antioxidants and anti-inflammatory capacity (Chiu and Taylor, 2007). It inhibits lipid peroxidation in the cell membrane (Traber and Atkinson, 2007). Carotene (α and β-carotene) (provitamin A) are terpenoids mainly found in the carrot, orange, mangoes, and papaya (Figure 1). They are involved in vision improvement and prevention of night blindness in humans (Strobel et al., 2007). SanGiovanni et al. (2007) reported that dietary intake of a combination of vitamin A, C and E in human study significantly decreased the AMD risk when compared to the individual vitamins. Similarly, Aoki et al. (2016) observed that supplementation of the mixture of A, C and E resulted in the inhibition of AMD. It was also observed that supplementation of provitamin A, vitamin C and E reduced the risk of neovascular AMD (Zampatti et al., 2014). However, there are no published or reported preventive or inhibitory effects of the individual vitamins (A, C, E) or provitamin A on the reviewed age-related eye diseases presented in this study (Table 1).

## 6 Conclusion

The reviewed dietary plant natural products (DPNPs) in this study have demonstrated strong preventive and therapeutic effects on age-related eye diseases such as age-related macular degeneration (AMD), cataract, diabetic retinopathy and glaucoma in both animal and human model experiments. The positive effects observed in DPNPs are due to their antioxidant, anti-inflammatory, and anti-angiogenic properties as well as their capacity to improve the antioxidant defence systems. Despite the promising preventive effects of DPNPs and documented scientific evidences against age-related eye diseases, there are no supportive documented approvals for their usage in the prevention, management and treatment of these diseases. This may be due to lack of controlled long term human clinical trials that will determine the effective doses of these DPNPs to be administered without any detrimental effects on humans. Therefore, further studies are needed to determine the optimal doses of individual DPNPs, the route of administration as well as their toxic doses before they can be recommended for use in the management, prevention or treatment of age-related eye diseases in both animals and humans.

## Declarations

### Author contribution statement

All authors listed have significantly contributed to the development and the writing of this article.

### Funding statement

This research did not receive any specific grant from funding agencies in the public, commercial, or not-for-profit sectors.

### Competing interest statement

The authors declare no conflict of interest.

### Additional information

No additional information is available for this paper.
