# Corrigendum to “The potential health benefits of dietary natural plant products in age related eye diseases” [Heliyon Volume 6, Issue 7, July 2020, Article e04408]

**DOI:** 10.1016/j.heliyon.2025.e43344

**Published:** 2025-05-02

**Authors:** Eleazar Uchenna Ikonne, Victor Okezie Ikpeazu, Eziuche Amadike Ugbogu

**Affiliations:** a Department of Optometry, Abia State University, P.M.B 2000, Uturu, Abia State, Nigeria; b Department of Biochemistry, Abia State University, P.M.B 2000, Uturu, Abia State, Nigeria

In this article, the authors provided **three** incorrect references ‘Kim et al., 2010’ (reference 51) Tesfaye et al., 2005 (reference 107) and Liu et al., 2017 (reference 64). The authors have provided the correct references that should be associated with the statements.

The original citations for ‘Kim et al., 2010’ in section ‘1. Introduction’ and ‘3.1 Age-related macular degeneration (AMD)’ alongside the reference can be found below:

***1. Introduction***

*“Loss of vision caused by age-related eye diseases has compromising effects on quality of life and may also result in an economic burden on the individual, the family and society (Kim* et al. *2010)”*

***3.1. Age-related macular degeneration (AMD)***

*“The major risk factors are age, arteriosclerosis, family history, hypertension, hypercholesterolemia, obesity and smoking (Kim et al. 2010).”*

The original citation for ‘Tesfaye et al., 2005’ in section ‘3.4. Diabetic retinopathy’ and the reference can be found below:

***3.4. Diabetic retinopathy***

*“Oxidative stress occurs because of imbalance between anti-oxidative mechanisms and reactive oxygen species, and this causes an increase in glucose levels (Tesfaye et al., 2005).”*

*Tesfaye, S., Chaturvedi, N., Eaton, S.E., Ward, J.D., Manes, C., Ionescu-Tirgoviste, C., Witte, D.R., Fuller, J.H., EURODIAB prospective complications study Group, 2005. Vascular risk factors and diabetic neuropathy. N. Engl. J. Med. 352 (4), 341–350.*

The original citations for ‘Liu et al., 2017’ in section ‘3.3 Cataract’ and the reference can be found below:

***3.3. Cataract***

*“In middle-income and low-income countries cataract is the leading cause of blindness (Liu et al., 2017) and 95 million individuals worldwide suffer cataract (Liu et al., 2017).”*

*“Clinically, cataracts can be classified into three types; cortical cataracts, nuclear cataracts and posterior subcapsular cataracts. However, it is important to note that the three types can associate with each other when left untreated as they progress to cause complete lens opacification (Liu et al., 2017).”*

*“Subcapsular cataract is a plaque-like opacity that emanates from the axial posterior cortical layer (Sparrow et al., 1986; Liu et al., 2017).”*

*Liu, Y., Liu, G., Cao, M., Chen, Q., Sun, L., Ji, B., 2017. Potential retinal benefits of dietary polyphenols based on their permeability across the blood-retinal barrier. J. Agric. Food Chem. 65 (15), 3179–3189.*

The incorrect citations and reference for ‘Kim et al., 2010’ should instead be associated with ‘Kim et al., 2019’. The incorrect citation and reference for ‘Tesfaye et al., 2005’ should instead be associated with ‘Tangvarasittichai, 2015’. The incorrect reference for ‘Liu et al., 2017’ should instead be associated with ‘Liu et al., 2017’ and as a result citations remain the same.

The correct version of the citations and references should be as below:

***Kim, C, Park, S., Youngji Kim, Y. 201*** ***8. Age-related macular degeneration among the elderly: The 5th National Health and Nutrition Examination Survey, 2010 through 2012. Jpn J Nurs Sci. 2019; 111.***

***2. Introduction***

*“Loss of vision caused by age-related eye diseases has compromising effects on quality of life and may also result in an economic burden on the individual, the family and society (Kim et al.* ***2019*** *)”*

***3.1. Age-related macular degeneration (AMD)***

*“The major risk factors are age, arteriosclerosis, family history, hypertension, hypercholesterolemia, obesity and smoking (Kim et al.* ***2019*** *).”*

***Tangvarasittichai S. (2015). Oxidative stress, insulin resistance, dyslipidemia and type 2 diabetes mellitus. World*** ***J*** ***ournal of*** ***D*** ***iabetes, 6(3), 456480.*** ***https://doi.org/10.4239/wjd.v6.i3.456***

***3.4. Diabetic retinopathy***

*“Oxidative stress occurs because of imbalance between anti-oxidative mechanisms and reactive oxygen species, and this causes an increase in glucose levels (*Tangvarasittichai, 2015*).”*

***Liu, Y. C., Wilkins, M., Kim, T., Malyugin, B., & Mehta, J. S. (2017). Cataracts. Lancet, 390(10094), 600–612.*** ***https://doi.org/10.1016/S0140-6736(17)30544-5*** ***.***

***3.3. Cataract***

*“In middle-income and low-income countries cataract is the leading cause of blindness (Liu et al., 2017) and 95 million individuals worldwide suffer cataract (Liu et al., 2017).”*

*“Clinically, cataracts can be classified into three types; cortical cataracts, nuclear cataracts and posterior subcapsular cataracts. However, it is important to note that the three types can associate with each other when left untreated as they progress to cause complete lens opacification (Liu et al., 2017).”*

*“Subcapsular cataract is a plaque-like opacity that emanates from the axial posterior cortical layer (Sparrow et al., 1986; Liu et al., 2017).”*

The ***authors*** apologize for the errors.

## Declaration of competing interest

The authors declare no conflict of interest.

